# Epithelial Tumors Originate in Tumor Hotspots, a Tissue-Intrinsic Microenvironment

**DOI:** 10.1371/journal.pbio.1002537

**Published:** 2016-09-01

**Authors:** Yoichiro Tamori, Emiko Suzuki, Wu-Min Deng

**Affiliations:** 1 Department of Biological Science, Florida State University, Tallahassee, Florida, United States of America; 2 Structural Biology Center, National Institute of Genetics and Department of Genetics, School of Life Science, The Graduate University for Advanced Studies (SOKENDAI), 1111 Yata, Mishima, Japan; Zentrum für Molekulare Biologie der Universität Heidelberg, GERMANY

## Abstract

Malignant tumors are caused by uncontrolled proliferation of transformed mutant cells that have lost the ability to maintain tissue integrity. Although a number of causative genetic backgrounds for tumor development have been discovered, the initial steps mutant cells take to escape tissue integrity and trigger tumorigenesis remain elusive. Here, we show through analysis of conserved neoplastic tumor-suppressor genes (nTSGs) in *Drosophila* wing imaginal disc epithelia that tumor initiation depends on tissue-intrinsic local cytoarchitectures, causing tumors to consistently originate in a specific region of the tissue. In this “tumor hotspot” where cells constitute a network of robust structures on their basal side, nTSG-deficient cells delaminate from the apical side of the epithelium and begin tumorigenic overgrowth by exploiting endogenous Janus kinase/signal transducer and activator of transcription (JAK/STAT) signaling activity. Conversely, in other regions, the “tumor coldspot” nTSG-deficient cells are extruded toward the basal side and undergo apoptosis. When the direction of delamination is reversed through suppression of RhoGEF2, an activator of the Rho family small GTPases, and JAK/STAT is activated ectopically in these coldspot nTSG-deficient cells, tumorigenesis is induced. These data indicate that two independent processes, apical delamination and JAK/STAT activation, are concurrently required for the initiation of nTSG-deficient-induced tumorigenesis. Given the conservation of the epithelial cytoarchitecture, tumorigenesis may be generally initiated from tumor hotspots by a similar mechanism.

## Abstract 2

A genetic study in *Drosophila* reveals a mechanism of tumorigenesis by which pro-tumor cells initiate dysplastic tumor growth at specific "hotspot" locations in epithelial tissues.

## Author Summary

Transformed mutant cells (pro-tumor cells) can evolve through a multistep process in which they become tumorigenic and invasive. Many genes that are involved in the different steps towards cancer development have been identified; however, how certain mutant cells destroy normal tissue organization and undergo uncontrolled proliferation during the initial stages of this process remains largely unclear. Using the epithelial tissue of the wing imaginal discs of the fruit fly (*Drosophila melanogaster*) larvae as a model system, we have analyzed these initial stages of inducing tumors by depletion of a neoplastic tumor suppressor gene (nTSG). We discovered that these tumors always originate from specific regions of the epithelial tissue of the wing disc. We show that in other regions that we dubbed “tumor coldspots” and that lack specific cellular structures, pro-tumor cells are eliminated from the epithelial tissue by the surrounding cells. However, in “tumor hotspots,” cells constitute specific structures in their basal side, and we found that pro-tumor cells successfully avoid potential elimination and deviate from the apical side of the tissue, initiating tumorous overgrowth. Our findings reveal the molecular and cellular mechanisms underlying the initial steps of tumorigenesis at tumor hotspots in the Drosophila imaginal wing discs.

## Introduction

In epithelial tissues, cells communicate with their neighbors and receive information from the surrounding environment through signaling networks, adhesion molecules, and junctional molecules in order to form complex organs and maintain their integrity and morphology. This robust, self-organizing system, however, is progressively disrupted during tumor development. In tumorigenesis, transformed mutant cells evolve into a malignant neoplasm through a multistep process whereby the transformed cells acquire traits that enable them to become tumorigenic and ultimately malignant [1]. Although many genes have been identified as involved in different steps of cancer cell progression, little is known about the beginning of tumorigenesis, in which only small subsets of mutant pro-tumor cells deviate from the robustly organized microenvironment to evolve into aggressive tumors.

Studies of a group of *Drosophila* tumor-suppressor genes, *lethal giant larvae* (*lgl*), *discs large* (*dlg*), and *scribble* (*scrib*), highlighted the critical relationship between loss of epithelial organization and tumor development. These genes play key roles in regulation of apical-basal cell polarity and cell proliferation in epithelial tissues [2]. In developing imaginal discs, epithelial tissues that are homozygous mutant for any of these three genes cause cells within the normally monolayered epithelia to lose structure and polarity, fail to differentiate, and overproliferate, thus becoming multilayered amorphous masses that fuse with adjacent tissues [2]. Similarly, loss or alteration in expression of these genes in mammals has been shown to be involved in development of malignant tumors [3,4]. The neoplastic phenotypes exhibited by the mutant tissues have led to the classification of these three genes as conserved neoplastic tumor-suppressor genes (nTSGs) [2,3]. However, when nTSG mutant fly cells are generated in otherwise wild-type epithelia using the FLP/FRT-mediated mitotic recombination technique, they undergo cell-competition-dependent apoptosis and are eliminated from the epithelial tissue [5–8]. This cell-competition-dependent elimination of nTSG-depleted cells has recently been confirmed in mammalian cells [9], suggesting cell competition is an evolutionarily conserved tissue-homeostatic mechanism that ensures elimination of pro-tumor cells and epithelial integration.

Here, we show that nTSG-deficient pro-tumor cells evade competitive pressure to take a first step toward evolving into aggressive tumors when they are located in a specific region within an epithelium and how this tissue-intrinsic local microenvironment has a decisive role for the life-or-death fate of pro-tumor cells.

## Results and Discussion

### nTSG-Knockdown Cells Undergo Site-Specific Tumor Growth in Wing Imaginal Discs

**Fig 1:**
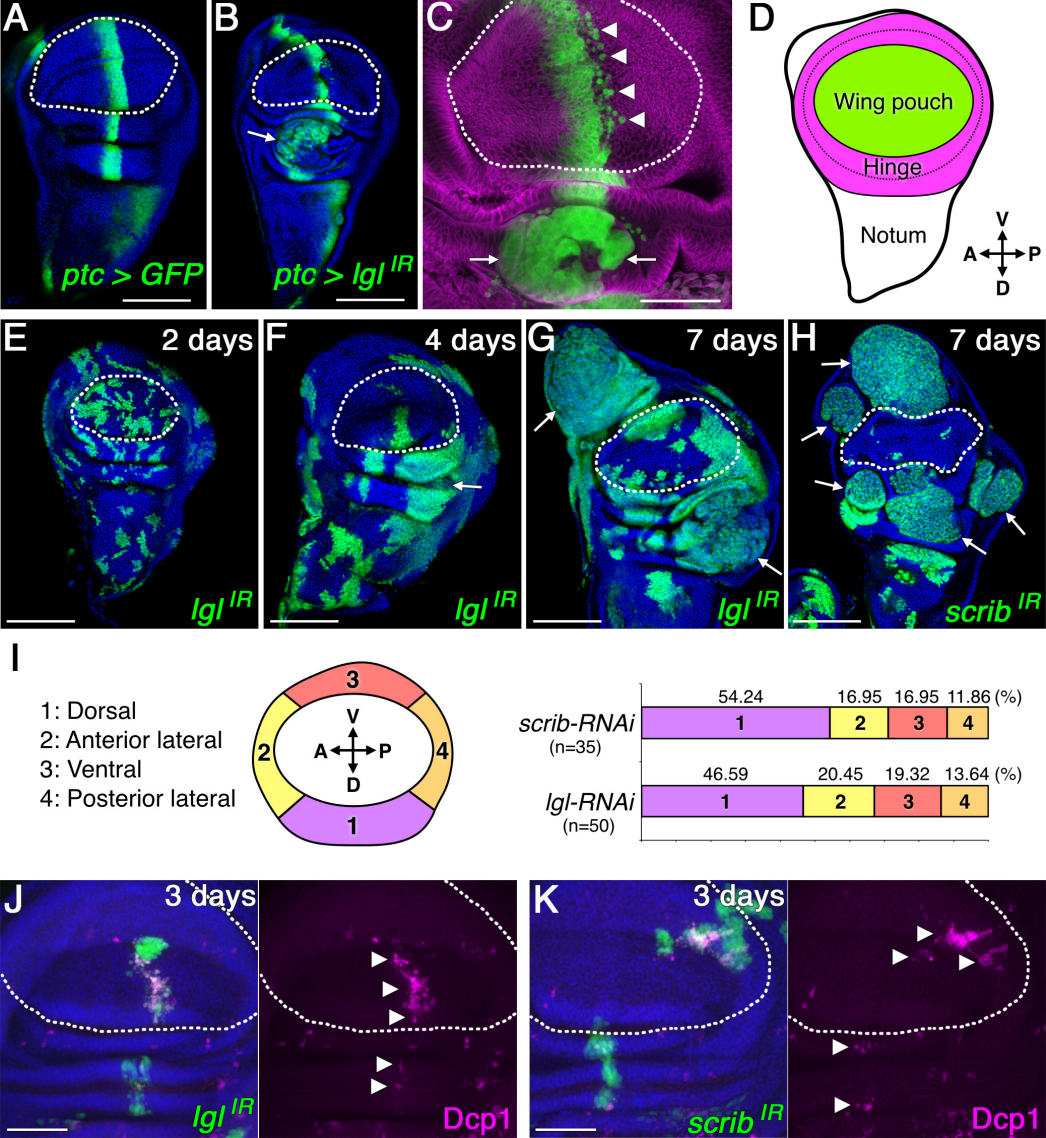
nTSG-knockdown cells induce site-specific tumor growths in wing imaginal discs. (A–C) Confocal images show wing imaginal discs dissected from indicated genotypes. *ptc-Gal4*-expressing regions were labeled by GFP expression (green). The disc was stained for α-tubulin (magenta) in (C). (D) Schematic representation of *Drosophila* wing imaginal discs showing wing pouch (green) and hinge (magenta) regions. (E–H) Mosaic wing discs with clones expressing *lgl-RNAi* (E–G) or *scrib-RNAi* (H) at the indicated time point after RNAi induction. RNAi-expressing cells were marked with GFP expression (green). (I) Quantified occurrence ratio of tumorigenesis induced by *scrib* or *lgl* knockdown in the hinge region. (J–K) Mosaic wing discs with clones expressing *lgl-RNAi* (J) or *scrib-RNAi* (K) at the indicated time point after RNAi induction. RNAi-expressing cells were marked with GFP expression (green). Apoptotic cells were labeled with anti-cleaved *Drosophila* Dcp-1 antibody (magenta). Nuclei were labeled with DAPI (blue) in (A–B), (E–H), and (J–K). White arrows indicate dysplastic tumor growths in (B–C) and (F–H). White arrowheads indicate apoptotic clones in (C) and (J–K). White dotted lines mark the boundaries between wing pouch and hinge regions in (A–C), (E–H), and (J–K). Scale bars represent 100 μm in (A–B) and (E–H) and 50 μm in (C) and (J–K).

Through close examination of nTSG-deficient cells in *Drosophila* wing imaginal discs, we found RNA interference (RNAi)-induced silencing of a single nTSG caused tumorigenic overgrowth in specific areas of the disc (Fig 1A and 1B). At the same time, many of the nTSG-knockdown cells adjacent to wild-type normal cells underwent cell-competition-induced apoptosis (Fig 1C, S1A and S1B Fig), as has been previously shown [6–8]. When *lgl* was knocked down by *ptc-Gal4-*driven *lgl-RNAi* along the anterior-posterior (AP) boundary of developing wing imaginal discs, dysplastic overgrowth was induced in the dorsal hinge region where the epithelial sheet is folded (Fig 1B, 1C and 1D). In contrast, the *ptc-Gal4*-induced misexpression of a constitutively active form of Yorkie (*YkiM123*), a transcriptional co-activator of the Hippo tumor-suppressor pathway, did not cause dysplastic overgrowth and instead showed hyperplastic overgrowth throughout the entire region of misexpression (S1C Fig). This clear phenotypic difference between *lgl* knockdown and Yki over-activation can be explained by their functions in two different tumor suppressor pathways; nTSGs are involved in epithelial organization through maintenance of apical-basal polarity [3] and mitotic spindle orientation [10–12], while the Hippo hyperplastic tumor suppressor pathway is involved in regulation of cell proliferation and apoptosis [13]. However, the difference between the phenotypes induced by *lgl* knockdown in the dorsal hinge and other regions in the same epithelial tissue was unexpected.

We therefore hypothesized that specific regions in the imaginal epithelial tissue are intrinsically susceptible to tumorigenic stimuli. To test the hypothesis of site-specific tumorigenesis, we generated random clones of nTSG-knockdown cells using the heat-shock-activated flip-out Gal4 system [14]. The dysplasia induced by *lgl* or *scrib* knockdown became clear in the hinge region 4 d after heat-shock-induced RNAi expression, and dysplastic tumor growths in the region were clearly observed 7 d after RNAi induction (Fig 1E–1H). These tumors showed strongly up-regulated expression of both phosphorylated c-Jun N-terminal kinase (pJNK) and matrix metalloproteinase-1 (MMP1) (S2A and S2B Fig), indicating an invasive cellular behavior [15]. In the hinge area, the occurrence ratio of nTSG-knockdown-induced dysplastic cell mass (each cell mass was counted as a single event of tumorigenesis) was the highest in the dorsal hinge (Fig 1I). In the wing pouch region, however, these nTSG-knockdown cells did not show dysplastic tumor growth (0%, *n* = 85). Apoptosis was detected in some nTSG-knockdown cells in both the wing pouch and hinge regions of the mosaic wing disc 3 d after RNAi induction (Fig 1J and 1K). These apoptotic cells, detected by cleaved Dcp-1 staining, were mostly at the boundary between the nTSG-knockdown cells and wild-type cells (81.7%, *n* = 278 apoptotic *lgl-*knockdown cells), suggesting cell competition is involved in eliminating nTSG-knockdown cells, as described previously [6–8]. Despite cell-competition-induced apoptosis, surviving nTSG-knockdown cells showed dysplastic tumor growth in the hinge region but not the pouch area. These results suggest the tumorigenic potential of pro-tumor cells with apical-basal polarity defects is dependent on their local environment in the epithelial tissue. We therefore refer to the wing pouch region where pro-tumor cells do not show dysplastic tumor growth as a “tumor coldspot,” whereas the hinge region where pro-tumor cells induce tumorigenesis is referred to as a “tumor hotspot.”

### Endogenous JAK/STAT Activity Is Required to Induce Tumorigenesis of nTSG-Knockdown Cells

**Fig 2:**
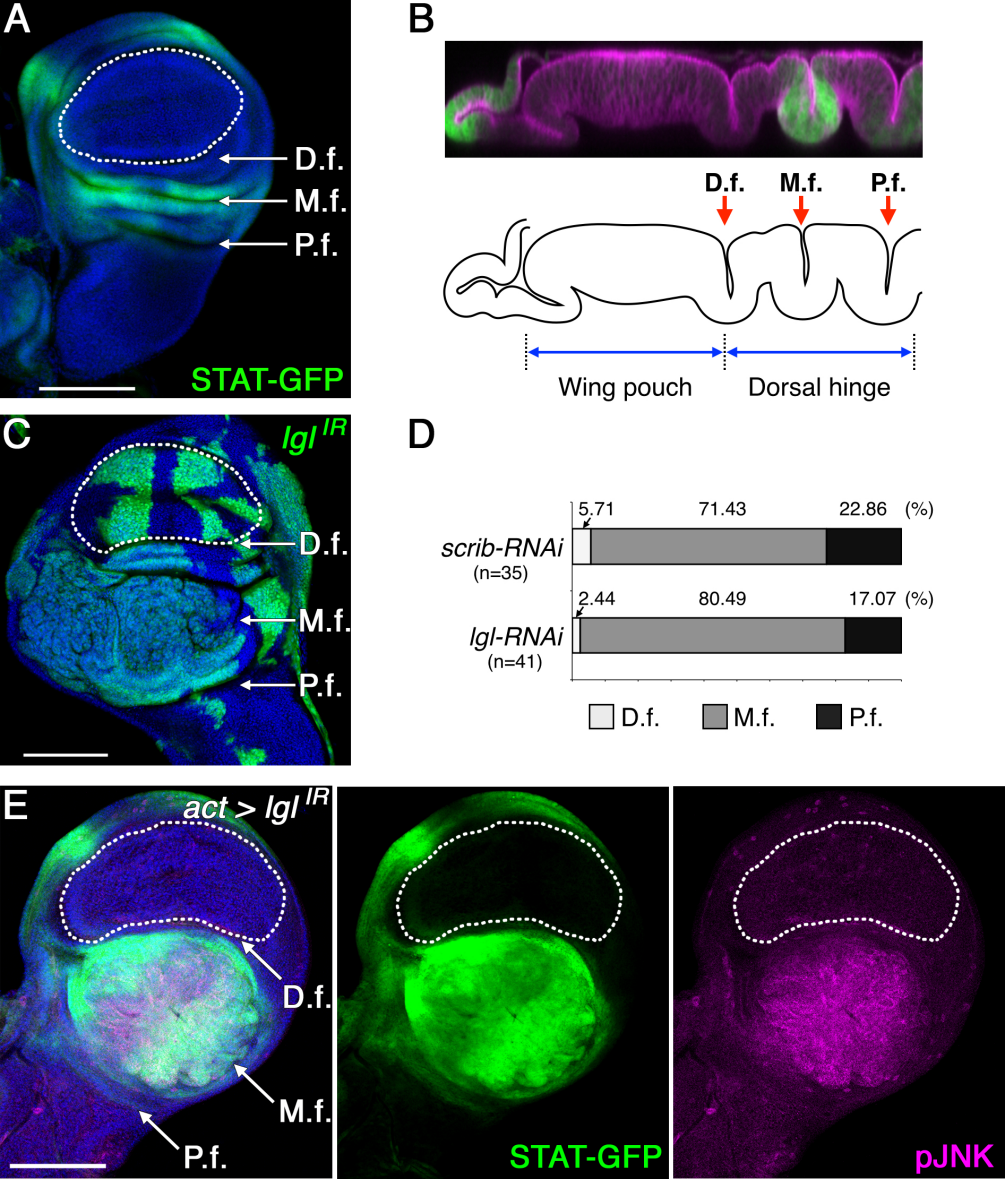
Endogenous JAK/STAT activity is required to induce tumorigenesis of nTSG-knockdown cells. (A) Normal wing imaginal disc with the JAK/STAT activity reporter, 10xSTAT-GFP (green). Distal (D.f.), medial (M.f.) and proximal (P.f.) folds of the dorsal hinge are indicated. (B) Vertical section along the AP boundary of a wing disc with 10xSTAT-GFP (green) stained for adherens junction component Armadillo (magenta) (top panel). Lower panel: black line drawings trace the apical and basal sides of the epithelial layer. (C) A mosaic wing disc with clones expressing *lgl-RNAi* and GFP 7 d after RNAi induction. (D) Quantified occurrence ratio of tumorigenesis induced by *scrib* or *lgl* knockdown in the dorsal hinge region. (E) pJNK staining (magenta) in a third instar wing disc with ubiquitous *lgl-RNAi* expression since early second instar. The expression of *lgl-RNAi* is spatiotemporally controlled by *act-Gal4* and temperature-sensitive (ts) Gal80 (Gal80^ts^). 10xSTAT-GFP, green. Nuclei were labeled with DAPI (blue) in (A), (C), and (E). White dotted lines mark the boundaries between the wing pouch and hinge regions in (A), (C), and (E). Scale bars represent 100 μm.

Tumorigenesis is normally associated with activation of growth-promoting signaling pathways [16], so we suspected the tumor hotspots in the wing disc might be related to local activation of these pathways. The Janus kinase/signal transducer and activator of transcription (JAK/STAT) pathway has been shown previously to be endogenously active in the hinge region of developing wing imaginal discs [17], and its dysregulation has been implicated in diverse types of human cancers [18] and in several tumor models in *Drosophila* [19,20]. Its secreted cytokine-like ligand Unpaired (Upd) [21], a *Drosophila* homolog of mammalian Interleukin-6 (IL-6), is endogenously expressed in dorsal, anterior lateral, and ventral posterior compartments of the hinge area [22]. The expression pattern of 10xSTAT92E-GFP, a JAK/STAT activity reporter, in wild-type tissues confirmed the endogenous activity of this pathway in these same tumor hotspots, particularly in the dorsal hinge region where its endogenous activity appears to be the highest (Fig 2A). The dorsal hinge has three epithelial folds: proximal, medial, and distal folds. The endogenous activity displayed by the JAK/STAT pathway is high in the medial fold, weak in the proximal fold, and barely detectable in the distal fold (Fig 2A and 2B) [23]. If tumorigenesis depends on JAK/STAT activity in the tumor hotspot, we predicted it would occur most obviously in the medial fold, where JAK/STAT activity is the highest. Indeed, the dysplastic tumor growth induced by *lgl*- or *scrib*-knockdown clones was mostly observed in the medial fold (Fig 2C and 2D). This site-specific tumorigenesis was further examined by suppressing nTSGs using different Gal4 drivers (*act-Gal4*, *sd-Gal4*), each of which has a broad expression pattern, including both the wing pouch and hinge regions in the wing disc (Fig 2E, S3A and S3B Fig). We found that the dysplastic tumor growth induced by these different drivers occurred most frequently in the medial fold of the dorsal hinge (71.19%, *n* = 59 tumors in dorsal hinge), with strong up-regulation of 10xSTAT-GFP in the tumorous tissue (Fig 2E). Conversely, no dysplastic tumor growth was detected in the wing pouch area where JAK/STAT signaling is inactive (0%, *n* = 59), and the nTSG-knockdown cells in this tumor coldspot did not show up-regulation of 10xSTAT-GFP (Fig 2E and S3C Fig). These findings suggest a strong correlation between endogenous JAK/STAT activation and tumorigenesis induced by nTSG knockdown in the wing imaginal disc.

**Fig 3:**
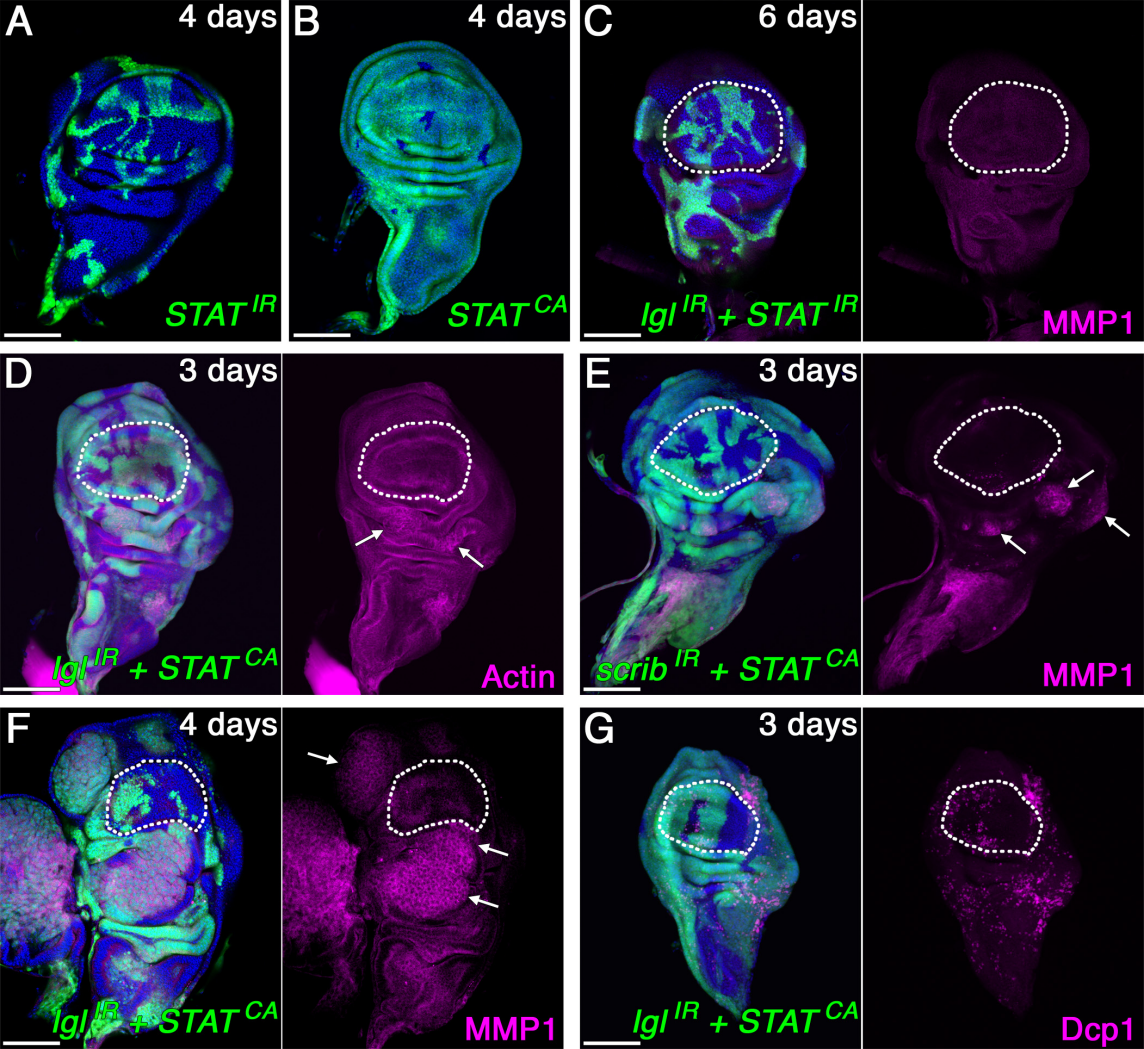
JAK/STAT activation is required yet not sufficient to induce tumorigenesis of nTSG-knockdown cells. (A) A wing disc with *STAT92E-RNAi* clones (co-expressing GFP, green) 4 d after clone induction. (B) A wing disc with *STAT92E*^*ΔNΔC*^ (the constitutively active STAT92E) expressing clones (green) 4 d after clone induction. (C) A wing disc with clones co-expressing *lgl-RNAi* and *STAT92E-RNAi* (green) 6 d after clone induction, stained for MMP1 (magenta). (D) A wing disc with clones co-expressing *lgl-RNAi* and *STAT92E*^*ΔNΔC*^ (green) 3 d after clone induction, stained for F-actin (magenta). (E) A wing disc with clones co-expressing *scrib-RNAi* and *STAT92E*^*ΔNΔC*^ (green) 3 d after clone induction, stained for MMP1 (magenta). (F) A wing disc with clones co-expressing *lgl-RNAi* and *STAT92E*^*ΔNΔC*^ (green) 4 d after clone induction stained for MMP1 (magenta). (G) A wing disc with clones co-expressing *lgl-RNAi* and *STAT92E*^*ΔNΔC*^ (green) 3 d after clone induction. Apoptotic cells were labeled with anti-cleaved *Drosophila* Dcp-1 antibody (magenta). White arrows indicate dysplastic tumor growth in (D–F). White dotted lines mark the boundaries between the wing pouch and hinge regions in (C–G). Nuclei were labeled with DAPI (blue). Scale bars represent 100 μm.

To determine whether JAK/STAT activation is required for tumor growth, we suppressed STAT92E, the *Drosophila* homolog of mammalian STAT3 and STAT5 [24,25], in *lgl-RNAi*-expressing mosaic discs by co-expression of *STAT92E*-*RNAi*. The mosaic wing disc bearing clones expressing *STAT92E-RNAi* alone did not show any obvious phenotype (Fig 3A). Depletion of STAT92E in *lgl*-knockdown cells, however, blocked the dysplastic tumor growth (Fig 3C), as has been shown in *scrib* and *dlg* mutant cells [26], indicating STAT activation is necessary for nTSG-knockdown-induced tumorigenesis. Next, we asked whether the activation of the JAK/STAT pathway is sufficient for the dysplastic tumor growth of nTSG-knockdown cells. To address this question, we ectopically activated JAK/STAT signaling in *lgl-RNAi*-expressing mosaic discs by co-expression of a constitutively active form of STAT92E (*STAT92E*^*ΔNΔC*^) [27]. First, we confirmed that ectopic expression of *STAT92E*^*ΔNΔC*^ without *lgl*- or *scrib*-knockdown showed no dysplastic tumor growth in wing discs (Fig 3B). However, over-activation of STAT in *lgl*- or *scrib*-knockdown cells in tumor hotspots, including the distal fold of the dorsal hinge, dramatically enhanced the tumor size (Fig 3D–3F) when compared with nTSG-knockdown-induced tumors without STAT over-activation from the same time point post-clone induction (Figs 1F and 3F). Three days after heat-shock induction, clones with co-expression of *lgl*- (or *scrib-*) *RNAi* and *STAT92E*^*ΔNΔC*^ showed clear dysplasia in the hinge regions (Fig 3D and 3E). This dysplasia developed into large tumor masses a day later (Fig 3F). In contrast, nTSG-knockdown cells without STAT over-activation only started to display dysplasia 4 d after clone induction (Fig 1F). To determine whether ectopic activation of STAT can transform a tumor coldspot into a hotspot, *STAT92E*^*ΔNΔC*^ was co-expressed with *lgl*- (or *scrib-*) *RNAi* in the pouch region. Still, tumors were not found in this coldspot, where apoptotic cell death was prevalent (Fig 3G). These results indicate that JAK/STAT activity is required yet not sufficient to induce tumorigenesis of nTSG-knockdown cells when they reside in tumor coldspots. Therefore, we hypothesized that additional factor(s) are responsible for the difference in tumorigenesis between coldspots and hotspots.

### Tumor Hotspots Have Structurally Robust Cellular Organizations in the Basal Side

**Fig 4:**
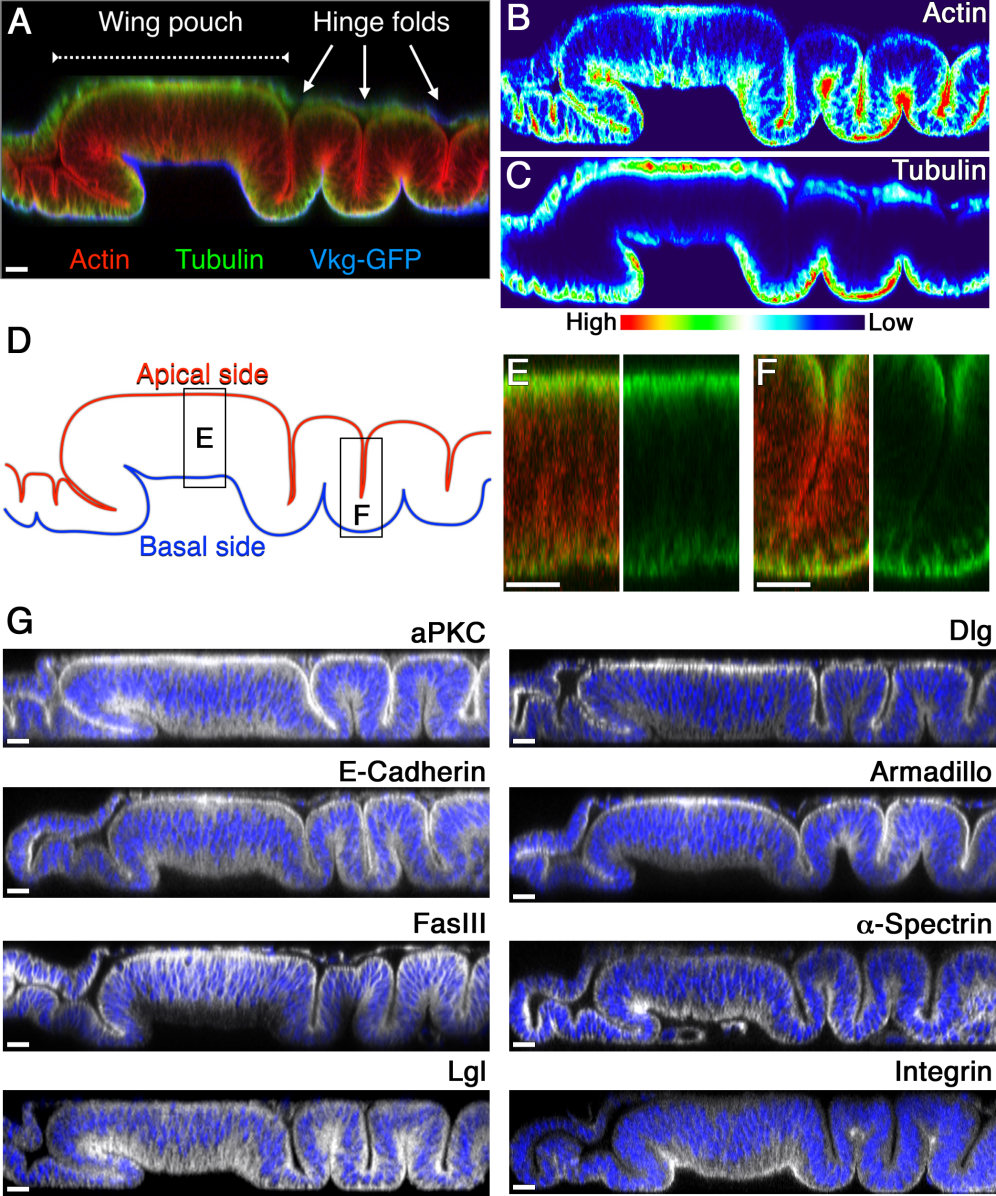
Subcellular localization of microtubules shows distinct pattern differences between the coldspot and hotspot. (A) Vertical section along the AP boundary of a wing disc labeled with a basement membrane marker Collagen IV/Vkg-GFP (blue). The disc was also stained for F-actin (red) and α-tubulin (green). (B–C) Signal intensities of actin (B) and α-tubulin (C) staining in (A) plotted using Interactive 3-D Surface Plot. (D) Line drawings trace the apical (red) and basal (blue) sides of the epithelial layer. (E–F) Magnified images of the wing pouch region (E) and the folded dorsal hinge region (F) indicated in (B). Left panels: merged images of F-actin (red) and α-tubulin (green) staining. Right panels: subcellular localization of α-tubulin (green). (G) Vertical sections along the AP boundary of wild-type wing discs were stained for the indicated proteins (white). Nuclei were labeled with DAPI (blue). Scale bars represent 10 μm.

One key difference between coldspots and hotspots is the morphology of columnar epithelial cells composing the pseudostratified monolayer, such that cells in the wing pouch (coldspot) have a long and straight shape along their apical-basal axis, whereas cells in the valley-folded hinge regions (hotspot) are shorter (Fig 4A). Therefore, we examined subcellular localization patterns of various proteins that function in cytoarchitectural construction of epithelial cells, which might serve to characterize the differences between morphologies. Measurement of proteins involved in the actin cytoskeleton (F-actin), components of the cell cortex (α-Spectrin and FasIII), apical complexes (aPKC), focal adhesion complexes (βPS-Integrin), adherens junctions (E-cadherin and Armadillo), and septate junctions (Dlg and Lgl) showed no significant differences in subcellular localization between the coldspot and hotspot (Fig 4A, 4B, and 4G). Interestingly, however, we found that intrinsic subcellular distribution of cortical microtubules (MTs), a cytoskeletal component crucial for the maintenance and modification of epithelial cell structures [28], showed distinct pattern differences between the coldspot and hotspot (Fig 4A and 4C–4F). MTs were localized primarily at the apical side of epithelial cells in the coldspot (Fig 4E). By contrast, the hotspot area lacked this apical enrichment of MTs. Instead, MTs appeared enriched at the basal side (Fig 4F).

**Fig 5:**
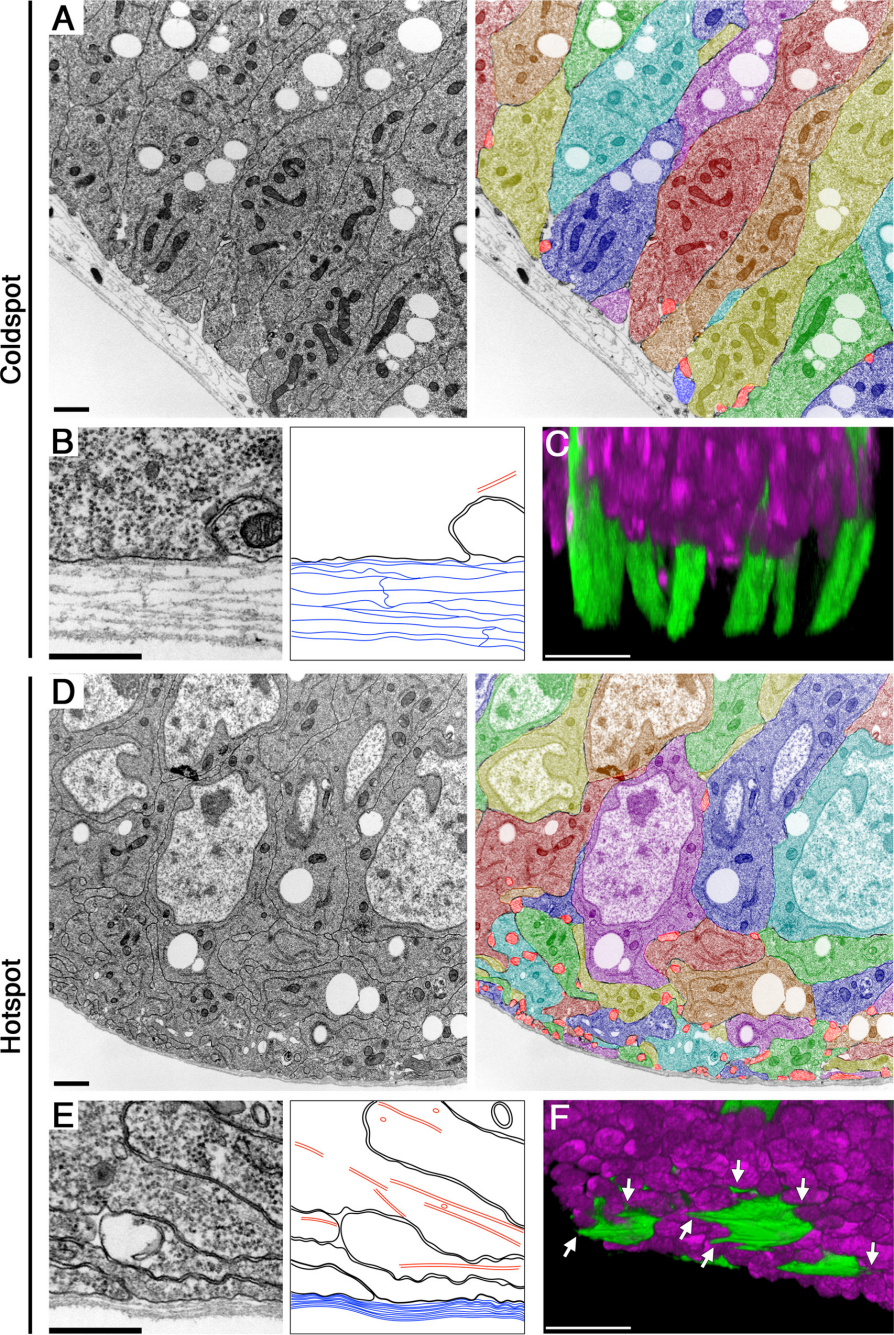
The basal side of the tumor hotspot has structurally robust cellular organizations. (A, D) TEMs showing the basal side of epithelial cells in the coldspot (A) and hotspot (D) of wild-type wing discs. Right panels of (A, D): cells in left panels were pseudo-colored individually to be distinguished from neighboring cells. Circular cross-sections (50–500 nm in radius) of filopodial protrusions were colored bright red. Scale bars represent 1 μm. (B, E) Transmission electron micrographs show magnified views of the most basal side of epithelial cells in the coldspot (B) and hotspot (E). Right panels of (B, E): line drawings trace the plasma membranes (black), ECM laminae (blue), and microtubules (red). Scale bars represent 500 nm. (C, F) Three-dimensional confocal images show the basal side of single cells randomly visualized by GFP expression (green) in the coldspot (C) and hotspot (F). Arrows indicate filopodial protrusions. Nuclei were labeled with DAPI (magenta). Scale bars represent 10 μm.

Furthermore, in transmission electron micrographs (TEM), we found other striking differences in cytoarchitecture between the hotspot and coldspot. The basement membrane, a sheet-like form of extracellular matrix (ECM) underlying epithelial tissues, is composed of approximately ten thin laminae in the imaginal epithelia. These ECM laminae were loosely organized in the coldspot, whereas they were tightly aligned in the hotspot (Fig 5A, 5B, 5D and 5E). Another remarkable difference was also identified in the basal side of the epithelial layer; in the valley-folded hotspot regions, cellular membranes displayed a complicated set of bends at the basal side, whereas in the coldspot they appeared straight along the apical-basal axis (Fig 5A and 5D). In addition, the TEM images revealed many circular-shaped double membrane structures at the basal side of the hotspot, but very few in the coldspot (Fig 5A and 5D). These circular membrane structures, which appeared similar to cross-sections of filopodial protrusions, varied in size (50–500 nm in radius) and had microtubules passing inside (Fig 5E). Indeed, when single cells were marked by randomly expressed membrane-GFP, laterally-elongated filopodial protrusions (5–10 μm) were consistently detected at the basal side of cells in the hotspot (Fig 5F). The web of protrusions appeared intricately intertwined among neighboring cells. In contrast, only shorter filopodial protrusions (1–5μm) were found at the basal side of coldspot cells (Fig 5C). Taken together, these observations suggest that the basal side of the hotspot contains robustly organized membrane and ECM structures, which may prevent delamination of pro-tumor cells from its surface.

### nTSG-Knockdown Cells Delaminate Apically and Undergo Tumor Growth in Hotspots

**Fig 6:**
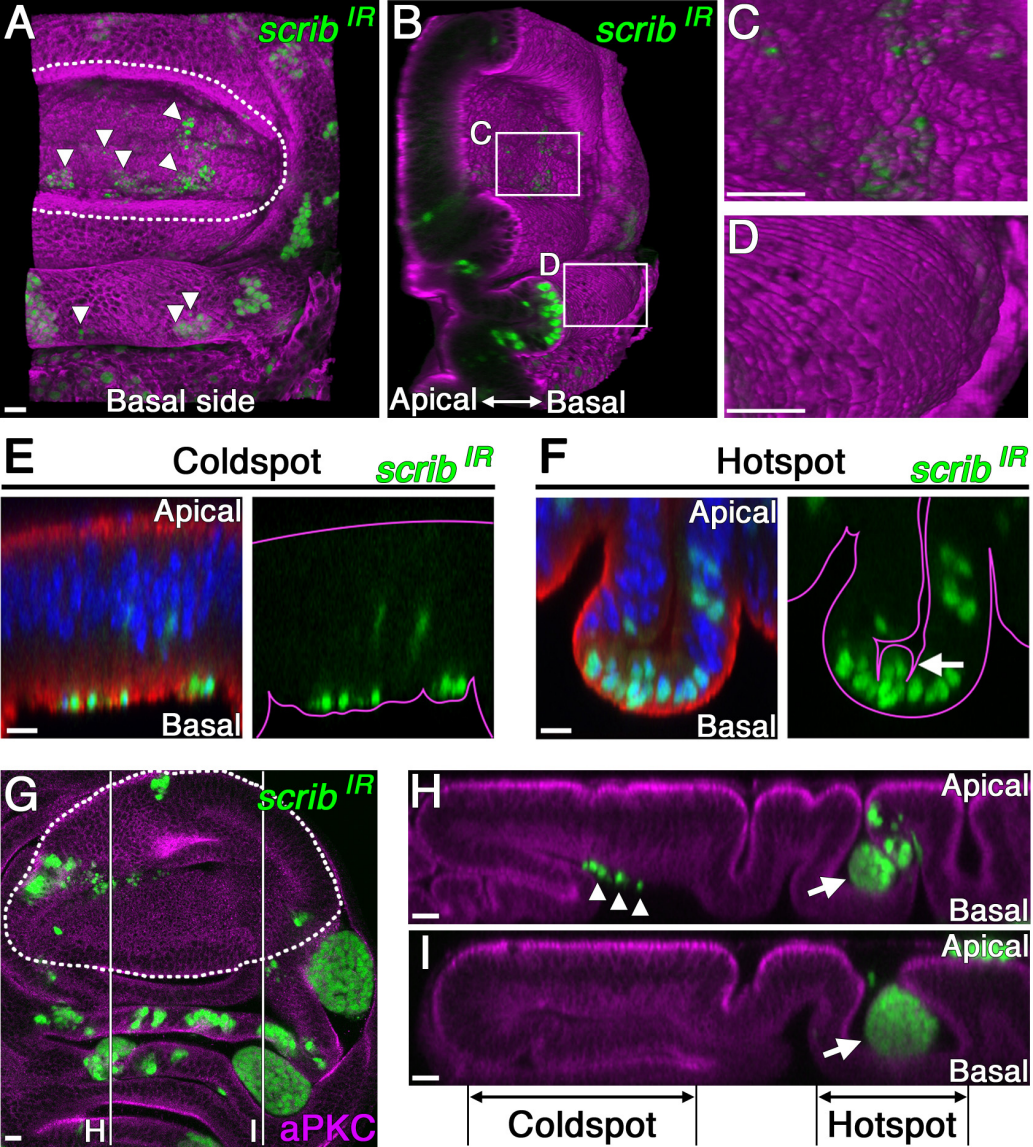
nTSG-knockdown cells delaminate apically and undergo tumor growth in hotspots. (A) Three-dimensional confocal image of a wing disc with clones co-expressing *scrib-RNAi* and GFP 2 d after clone induction stained for α-tubulin (magenta). The image shows a basal view of the disc. (B) Tilted images of (A) to visualize the extruded cells from the basal side of the epithelial layer. (C–D) Magnifications of the boxes indicated in (B). Extrusion of apoptotic *scrib*-knockdown cells (GFP marked, green) from the basal side was found in the coldspot (C), but not in the hotspot (D). (E–F) Vertical sections of indicated regions in a wing disc with clones co-expressing *scrib-RNAi* and GFP 2 d after clone induction, stained for α-tubulin (red). Nuclei were labeled with DAPI (blue). Right panels: magenta line drawings trace the apical and basal sides of the epithelial layer. Arrow in (F) indicates apically delaminating cells. (G) A wing disc with clones co-expressing *scrib-RNAi* and GFP 5 d after clone induction, stained for aPKC (magenta). (H–I) Vertical sections of two different sites marked with white lines in (G). Arrows indicate tumors in the apical side. Arrowheads indicate basally extruded cells. White dotted lines mark the boundaries between the wing pouch and hinge regions in (A) and (G). Scale bars represent 10 μm.

The aforementioned morphological observations led us to examine the early-phase behavior of pro-tumor cells in different regions of the wing disc through three-dimensional confocal imaging. In the wing pouch, the *lgl-RNAi*- or *scrib-RNAi*-expressing cells underwent apoptosis (Fig 1J and 1K) and were extruded from the basal side of the epithelial layer 2 d after clone induction (Fig 6A–6C). Apoptotic nTSG-knockdown cells were similarly observed in the basal side of the folded hinge hotspot region, yet remained within the epithelial layer (Fig 6A, 6B and 6D). These results suggest that apoptotic pro-tumor cells, found in both coldspot and hotspot regions, tend to be extruded toward the basal side of epithelial sheets, likely by myosin-II-dependent pulling forces generated by apoptotic cells, as has been previously shown [29]. Consistent with this observation, sporadic wing-disc cells misexpressing a pro-apoptotic gene, *Reaper*, were also extruded toward the basal side in both coldspots and hotspots (S4 Fig).

Interestingly, we found some surviving nTSG-knockdown cells in the hotspot delaminated from the apical side of the epithelial layer (Fig 6F). In contrast, no apical delamination of the nTSG-knockdown cells was detected in the coldspot (Fig 6E and 6H), consistent with the previous report that mitotic spindle misorientation caused by nTSG knockdown induces live-cell delamination from the basal side in the wing pouch. These basally delaminated cells then undergo apoptosis [10]. In the hotspot, by contrast, apically-delaminated cells appeared to have proliferated 5 d after clone induction, forming an amorphous mass of cells at the apical side (Fig 6G–6I). Indeed, the dysplastic tumor growth induced by nTSG knockdown always occurred at the apical side of the epithelial layer (100%, *n* = 127).

### Both Apical Delamination and Endogenous JAK/STAT Activity Are Required for nTSG Depletion-Induced Tumorigenesis

**Fig 7:**
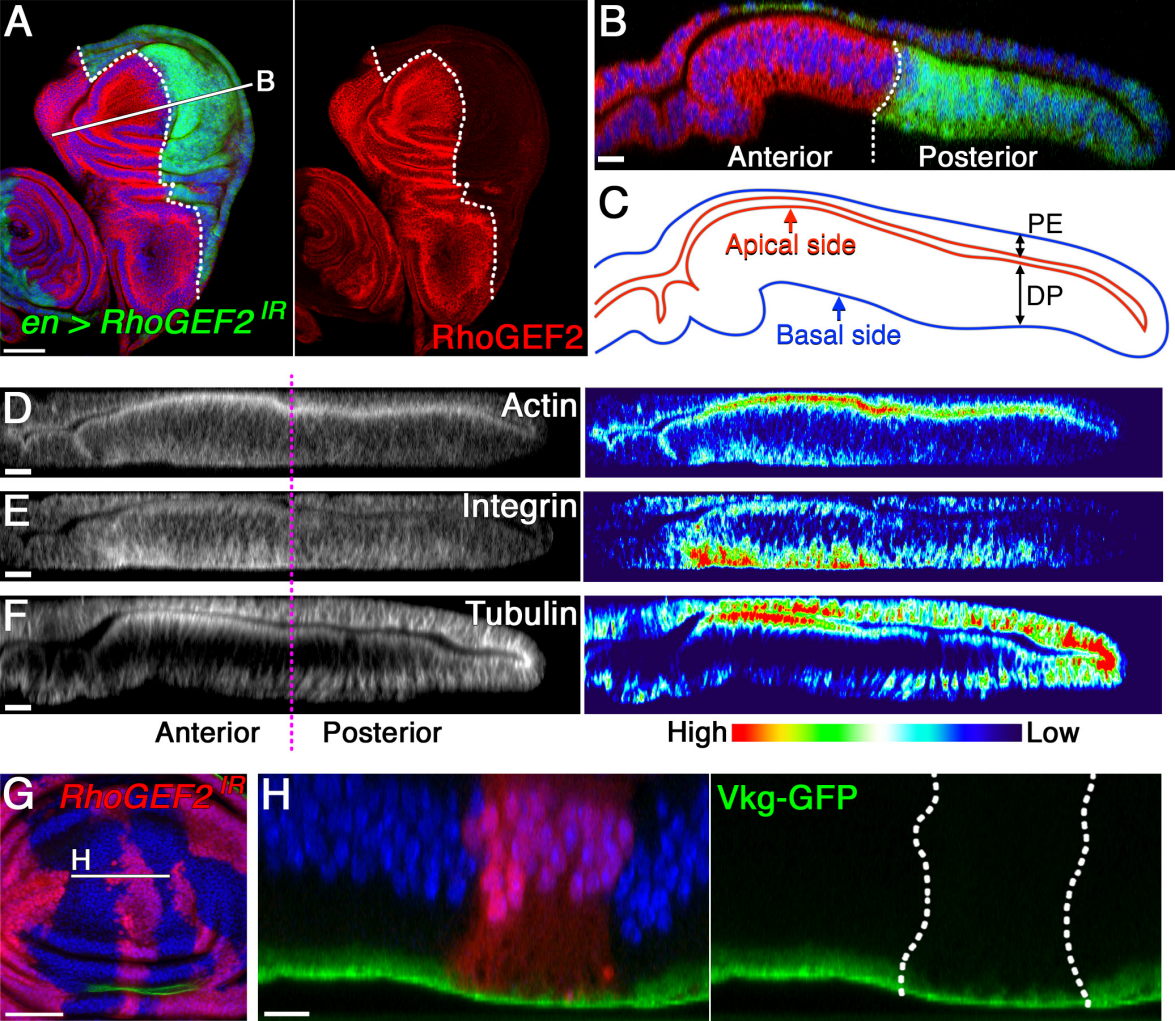
RhoGEF2 knockdown induces hotspot-like cytoarchitectures in the coldspot. (A) A wing disc of a third instar with *RhoGEF2-RNAi* expression in the posterior compartment (marked with GFP expression, green), stained for RhoGEF2 (red). Right panel shows *RhoGEF2* knockdown in the posterior compartment of the disc. A white dotted line marks the boundary between the anterior (left) and posterior (right) compartments. (B) A vertical section of the wing disc at a site marked with a white line in (A). (C) Line drawings trace the apical (red) and basal (blue) sides of the epithelial layer. PE, peripodial epithelium. DP, disc proper epithelium. (D–F) Left panels: vertical sections along the dorsal-ventral boundary of wing discs with *RhoGEF2-RNAi* expression in the posterior compartment (right side of magenta dotted line) stained for F-actin (D), βPS-Integrin (E), and α-tubulin (F) (white). Right panels: signal intensities plotted using Interactive 3-D Surface Plot. (G) A wing disc with clones (marked with RFP expression, red) expressing *RhoGEF2-RNAi* 4 d after clone induction. The basement membranes were labeled with Collagen IV GFP protein trap (Vkg-GFP), green. (H) A vertical section of the wing disc at a site marked with a white line in (G). White dotted lines mark the clonal boundaries. Nuclei were labeled with DAPI (blue) in (A), (B), and (G–H). Scale bars represent 50 μm in (A) and (G) and 10 μm in (B) and (D–F).

As has been described above, the tumorigenic overgrowth was detected most frequently in the medial fold where JAK/STAT activity was high (Fig 2D), suggesting apically delaminating pro-tumor cells are exposed to endogenous JAK/STAT signaling activity that is specifically activated in the medial fold for their survival and growth. Similar to reports in mammalian epithelial cells [30,31], *Drosophila* Upd is secreted from the apical surface of epithelial cells to transduce the signal to the neighboring cells, where it binds the receptor Domeless, which is also localized on the apical membrane [32]. Therefore, apical delamination in the valley-folded hotspot where the JAK/STAT ligand abundantly accumulates favors the survival and proliferation of the pro-tumor cells. If this hypothesis is true, we predicted that nTSG-knockdown cells delaminated apically would not undergo tumorigenesis in the coldspot where JAK/STAT signaling is not active. The guanine nucleotide exchange factor RhoGEF2, an activator of the Rho family small GTPases [33,34] has been implicated in apical constriction and the cell-shape change of epithelial cells during gastrulation [35,36], actomyosin contraction during blastoderm cellularization [37,38], and segmental groove formation during embryogenesis [39] in *Drosophila*. Furthermore, knockdown of *p115 RhoGEF*, a mammalian homolog of *RhoGEF2*, has been shown to disrupt the apically directed extrusion of apoptotic cells in cultured epithelial monolayers from human bronchia [40]. Therefore, we examined whether a defect in RhoGEF2 activity could disturb the delamination direction of nTSG-knockdown cells in the wing imaginal disc. Using the *en-Gal4* driver to knockdown *RhoGEF2* in the posterior compartment of a wing disc (Fig 7A–7C), we found that both apical enrichment of F-actin and basal enrichment of βPS-integrin were decreased in the pouch cells (Fig 7D and 7E). Unexpectedly, *RhoGEF2* knockdown also reversed the subcellular localization of cortical MTs in the coldspot: MTs became enriched at the basal side, opposite to their normal apical localization in the same region (Fig 7F). Moreover, the thickness of basement membranes visualized with a functional GFP-tagged Collagen IV (Vkg-GFP) was strongly reduced by *RhoGEF2* knockdown in the coldspot (Fig 7G and 7H) where ECM laminae were loosely organized (Fig 5A and 5B). These observations suggest that *RhoGEF2* knockdown induces hotspot-like cytoarchitectures in the coldspot area.

**Fig 8:**
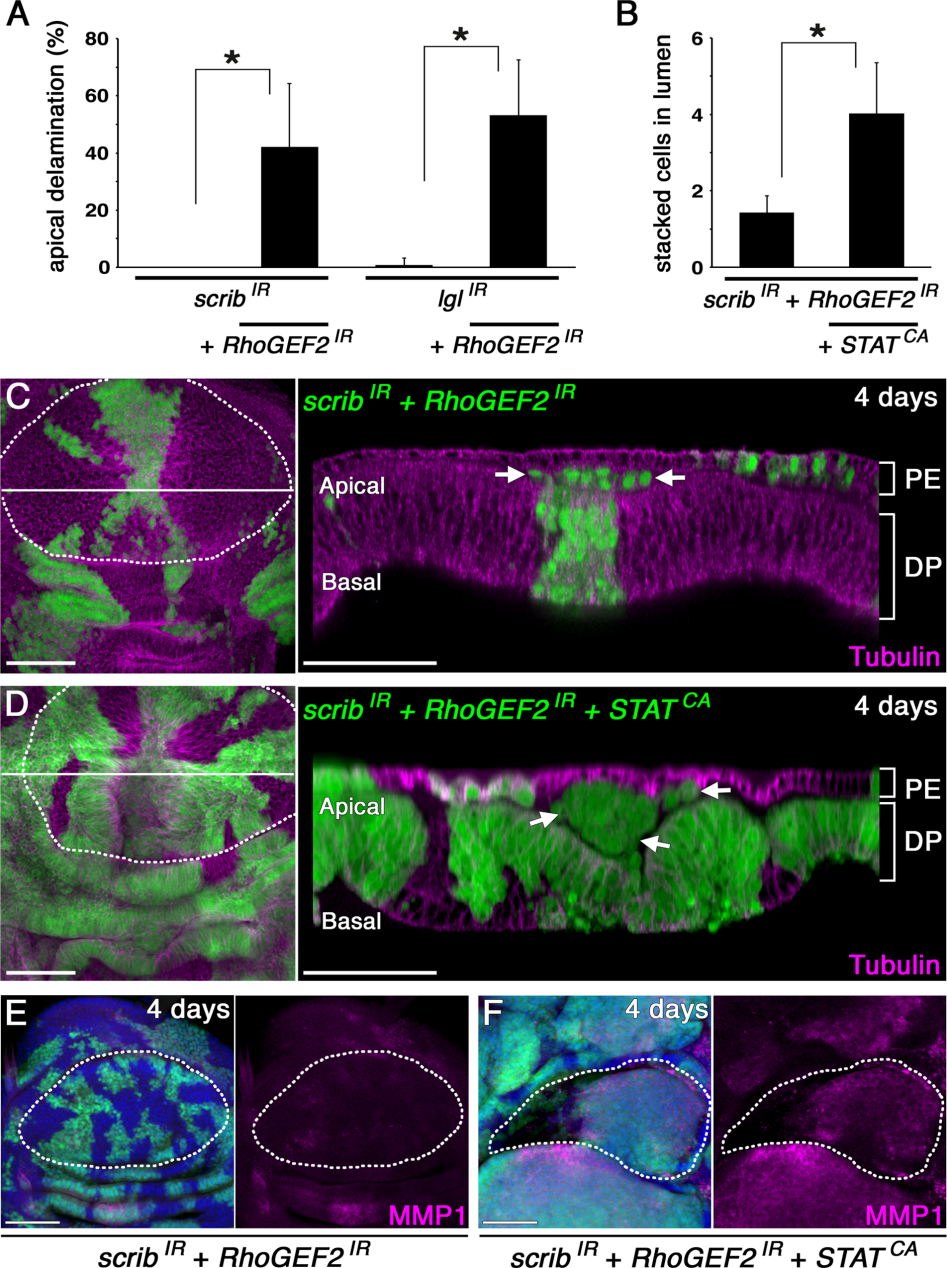
Apical delamination and STAT over-activation together promote nTSG-knockdown cells undergoing tumorigenesis in the coldspot. (A) Quantification of apical delamination in mosaic clones expressing *scrib-RNAi* (*n* = 60 clones from 10 wing discs), *scrib-RNAi*, and *RhoGEF2-RNAi* (*n* = 70 clones from 10 wing discs), *lgl-RNAi* (*n* = 71clones from 10 wing discs), or *lgl-RNAi* and *RhoGEF2-RNAi* (*n* = 62 clones from 10 wing discs) (from left to right). Means ± standard deviation (s.d.) of three independent experiments, *p < 0.001. (B) Size quantification of apically delaminated cell mass measured by number of stacked cells in the lumen of coldspots. *n* = 41 clones co-expressing *scrib-RNAi* and *RhoGEF2-RNAi* from 10 wing discs and 32 clones co-expressing *scrib-RNAi*, *RhoGEF2-RNAi*, and *STAT92E*^*ΔNΔC*^ from 10 wing discs. Means ± s.d. of three independent experiments, *p < 0.001. (**C**) Left panel: a wing disc with clones (marked with GFP expression, green) co-expressing *scrib-RNAi* and *RhoGEF2-RNAi* 4 d after clone induction, stained for α-tubulin (magenta). Right panel: a vertical section of the wing disc at a site marked with a white line in the left panel. (D) Left panel: a wing disc with clones (marked with GFP expression, green) co-expressing *scrib-RNAi*, *RhoGEF2-RNAi*, and *STAT92E*^*ΔNΔC*^ 4 d after RNAi induction, stained for α-tubulin (magenta). Right panel: a vertical section of the wing disc at a site marked with a white line in the left panel. Arrows indicate apically delaminated clones in the disc lumen. PE, peripodial epithelium. DP, disc proper epithelium. (E) A wing disc with clones (marked with GFP expression, green) co-expressing *scrib-RNAi* and *RhoGEF2-RNAi* 4 d after clone induction stained for MMP1 (magenta). (**F**) A wing disc with clones (marked with GFP expression, green) co-expressing *scrib-RNAi*, *RhoGEF2-RNAi*, and *STAT92E*^*ΔNΔC*^ 4 d after RNAi induction stained for MMP1 (magenta). Nuclei were labeled with DAPI (blue) in (E–F). White dotted lines in (C–F) mark the boundaries between the wing pouch and hinge regions. Scale bars represent 50 μm. The underlying numerical data for Fig 8A and 8B can be found in S1 Data.

To determine whether *RhoGEF2* knockdown has an effect on the delamination direction of nTSG-deficient cells in the disc, we co-knocked down *RhoGEF2* in *scrib-RNAi*- or *lgl-RNAi*-expressing mosaic discs. Consequently, loss of RhoGEF2 resulted in apical delamination of both *scrib-* and *lgl*-knockdown cells in the coldspot (Fig 8A and 8C). Because nTSG-knockdown alone resulted in only basal delamination in this same region (Figs 6H and 8A), we conclude that depletion of RhoGEF2 can disturb the delamination direction of nTSG-deficient cells. However, these apically delaminated *scrib*-knockdown cells did not show tumorigenic overgrowth in the lumen of coldspots 4 d after clone induction (Fig 8C). Since endogenous JAK/STAT signaling is required for tumorigenesis in the hotspot (Figs 2 and 3C), we asked whether ectopic activation of JAK/STAT signaling can induce tumorigenic overgrowth of nTSG-deficient cells delaminated apically at the coldspot. To this end, we misexpressed *STAT92E*^*ΔNΔC*^ together with *scrib-RNAi* and *RhoGEF2-RNAi* and found that *scrib*-knockdown cells with *RhoGEF2* suppression and STAT ectopic-activation showed apical delamination and tumor growth in the lumen of coldspots 4 d after clone induction (Fig 8B and 8D). These tumors showed strongly up-regulated expression of MMP1 (Fig 8E), which was not detected in *scrib*- and *RhoGEF2-*co-knockdown cells located in the coldspot (Fig 8D). These results indicate that both apical delamination and JAK/STAT activity are required for nTSG depletion-induced tumorigenesis in this *Drosophila* epithelial tissue.

Ectopic activation of STAT by *STAT92E*^*ΔNΔC*^ alone did not change the epithelial cytoarchitecture such as MT subcellular distribution in epithelial cells (S5A Fig). It did not change the extrusion direction of *scrib*-knockdown cells in the coldspot either (S5B Fig). On the other hand, *RhoGEF2* knockdown did not cause a change of JAK/STAT signaling activity in the disc (S5C Fig). These data suggest that the effects of each modification on nTSG-knockdown pro-tumor cells are discrete and that collaboration of these two independent processes (apical delamination and JAK/STAT activation) is required for the initiation of nTSG-depletion-induced tumorigenesis. Considering that ectopic activation of STAT alone in basally extruded nTSG-knockdown cells did not induce tumorigenesis in the coldspot, apical delamination provides the pro-tumor cells with a crucial survival advantage. Apoptosis of pro-tumor cells, such as nTSG mutant cells, is known to be induced by Eiger, the *Drosophila* homolog of mammalian tumor necrosis factor (TNF)-α, which is produced by circulating hemocytes recruited to tumor tissues [41]. Because hemocytes directly associate with cells along the basal side of the epithelial layer [42], apical delamination could have prevented the pro-tumor cells from receiving the death signal.

Our data describe how two different causal factors are closely involved in initiation of nTSG defect-induced tumorigenesis in tumor hotspots: apical delamination of surviving pro-tumor cells and high levels of endogenous JAK/STAT activity. The different sensitivity to tumorigenic growth between the distal pouch and proximal hinge regions in *Drosophila* wing imaginal discs has been reported in some previous studies [8,43,44]. However, the tumors induced by *lgl* mutant clones in those studies were created under specific experimental conditions in which the competitive pressure was removed or attenuated by conferring oncogenic activity on *lgl* mutant clones via overexpression of oncogenic Ras^V12^ or Yki, or lowering competitiveness of the surrounding cells via the *Minute* technique. In contrast, the site-specific tumorigenesis phenotype reported here was induced by knockdown of an nTSG alone, revealing an important role played by the endogenous local microenvironment on determining where tumors can grow. The tumor hotspots display a network of robust basal structures, including a web of intertwining filopodia and tightly laminated basement membranes, which could force pro-tumor cells to delaminate from the apical side and enter the lumen formed by the disc epithelium and peripodial membrane. In the disc lumen where JAK/STAT activity is high, such as the medial fold, tumorigenesis takes place in this niche-like environment. The cells in the medial fold of the dorsal hinge region also show intrinsic resistance to ionizing radiation (IR)- and drug-induced apoptosis and help tissue regeneration, further suggesting the intrinsic tumor susceptibility of this subpopulation [45]. A recent in vitro study using organotypic human mammary acini has shown apical translocation of single cells from the epithelial layer allows the oncogenic mutant cells to undergo luminal outgrowth [46]. In addition, chronic inflammation is linked to all stages of tumor development. It is well known that activation of STAT family members (particularly STAT3), which are closely linked to inflammatory processes in multiple tissues, is implicated in tumorigenesis, promoting proliferative and survival signaling [47]. In pathological histology, it is also known that tumors frequently arise in transitional zones where two different types of epithelial tissue meet, resulting in the appearance of a distinct abrupt transition, which can be found in numerous locations within various tissues [48]. For example, tumors in the anal canal were found to arise primarily in a transitional zone between stratified squamous epithelium of anal skin and mucosal epithelium of the large intestine [49]. Intriguingly, even in wild-type mice, the epithelium of the transitional zone in the anus intrinsically shows many features reminiscent of hyperproliferative epidermis including aberrant expression of differentiation markers, enhanced Ras-MAPK signaling, and locally increased inflammation [49,50]. Future studies will determine if the transitional zones in mammalian tissues have the tumor-hotspot-specific cytoarchitectures that we found in the *Drosophila* imaginal discs. Besides the transitional zones, however, it is also likely that tumor-hotspot microenvironments are easily formed by small alterations in epithelial cytoarchitectures. Given the widely conserved cytoarchitectures of metazoan epithelia, disruption of tissue organization initiated from intrinsic tumor hotspots could explain a general mechanism of tumorigenesis.

## Materials and Methods

### Fly Stocks and Genetics

*Drosophila* stocks were maintained by standard methods at 25°C. For generation of mosaic UAS-transgene overexpression clones in imaginal discs, first instar larvae (48 h after egg deposition) were heat-shocked for 15–60 min at 37°C. To control the timing of UAS-transgene overexpression with the temperature-sensitive *Gal80* (*Gal80ts*) in imaginal discs, fly larvae were kept in 18°C until they reached second instar stage and were then transferred to 29°C. The following fly strains were used: *ptc-Gal4* (Bloomington #2017), *sd-Gal4* (Bloomington #8609), *en-Gal4* (Bloomington #30564), *act-Gal4* (Bloomington #4414), *UAS-RhoGEF2-RNAi* (Bloomington #34643), *UAS-rpr* (Bloomington #5823), *UAS-lgl-RNAi* (VDRC #51247), *UAS-scrib-RNAi* (VDRC #105412), *UAS-STAT92E-RNAi* (VDRC #106980), *UAS-Yki*^*M123*^ [51], *UAS-3HA-STAT92E*^*ΔNΔC*^ [27], *10xSTAT-GFP* [22], and *vkg-GFP* (G00454). All genotypes of flies used in each experiment are described below.

### Detailed Genotypes for Each Experiment

Fig 1:

**A,** w; *ptc-Gal4*, *UAS-EGFP/+*
**B–C,** w; *UAS-dicer2/ptc-Gal4*, *UAS-EGFP; UAS-lgl-RNAi/+*
**E–G,** *hsFLP; UAS-dicer2/+; act>CD2>Gal4*, *UAS-GFP/UAS-lgl-RNAi*
**H,** *hsFLP; UAS-scrib-RNAi/+; act>CD2>Gal4*, *UAS-GFP/UAS-dicer2*
**I,** *hsFLP; UAS-dicer2/+; act>CD2>Gal4*, *UAS-GFP/UAS-lgl-RNAi*
*hsFLP; UAS-scrib-RNAi/+; act>CD2>Gal4*, *UAS-GFP/UAS-dicer2*
**J,** *hsFLP; UAS-dicer2/+; act>CD2>Gal4*, *UAS-GFP/UAS-lgl-RNAi*
**K,** *hsFLP; UAS-scrib-RNAi/+; act>CD2>Gal4*, *UAS-GFP/UAS-dicer2*

Fig 2:

**A–B,** w; *10xSTAT-GFP/+*
**C,** *hsFLP; UAS-dicer2/+; act>CD2>Gal4*, *UAS-GFP/UAS-lgl-RNAi*
**D,** *hsFLP; UAS-dicer2/+; act>CD2>Gal4*, *UAS-GFP/UAS-lgl-RNAi*
*hsFLP; UAS-scrib-RNAi/+; act>CD2>Gal4*, *UAS-GFP/UAS-dicer2*
**E,** *w; act-Gal4*, *UAS-GFP/UAS-dicer2; tubP-Gal80ts/UAS-lgl-RNAi*

Fig 3:

**A,** *hsFLP; UAS-STAT92E-RNAi/UAS-dicer2; act>CD2>Gal4*, *UAS-GFP/+*
**B,** *hsFLP; act>CD2>Gal4*, *UAS-GFP/+; UAS-3HA-STAT92E*^*ΔNΔC*^*/+*
**C,** *hsFLP; UAS-STAT92E-RNAi/UAS-dicer2; act>CD2>Gal4*, *UAS-GFP/UAS-lgl-RNAi*
**D,** *hsFLP; UAS-3HA-STAT92E*^*ΔNΔC*^*/UAS-dicer2; act>CD2>Gal4*, *UAS-GFP/UAS-lgl-RNAi*
**E,** *hsFLP; act>CD2>Gal4*, *UAS-GFP/UAS-scrib-RNAi; UAS-3HA-STAT92E*^*ΔNΔC*^*/UAS-dicer2*
**F,** *hsFLP; UAS-3HA-STAT92E*^*ΔNΔC*^*/UAS-dicer2; act>CD2>Gal4*, *UAS-GFP/UAS-lgl-RNAi*
**G,** *hsFLP; UAS-3HA-STAT92E*^*ΔNΔC*^*/UAS-dicer2; act>CD2>Gal4*, *UAS-GFP/UAS-lgl-RNAi*

Fig 4:

**A–F,** yw; *vkg-GFP/+*
**G,** *w*

Fig 5:

**A–B,** *w*
**C,** *hsFLP;; act>CD2>Gal4*, *UAS-GFP/+*
**D–E,** *w*
**F,** *hsFLP;; act>CD2>Gal4*, *UAS-GFP/+*

Fig 6:

**A–I,** *hsFLP; UAS-scrib-RNAi/+; act>CD2>Gal4*, *UAS-GFP/UAS-dicer2*

Fig 7:

**A–F,** *w; en-Gal4*, *UAS-EGFP/+; UAS-RhoGEF2-RNAi/+*
**G–H,** *hsFLP; Vkg-GFP/+; act>CD2>Gal4*, *UAS-RFP/UAS- RhoGEF2-RNAi*

Fig 8:

**A,** *hsFLP/UAS-dicer2; UAS-scrib-RNAi/+; act>CD2>Gal4*, *UAS-GFP /UAS-RhoGEF2-RNAi*
**B,** *hsFLP/UAS-dicer2; act>CD2>Gal4*, *UAS-GFP/UAS-scrib-RNAi; UAS-3HA-STAT92E*^*ΔNΔC*^*/UAS-RhoGEF2-RNAi*
**C,** *hsFLP/UAS-dicer2; UAS-scrib-RNAi/+; act>CD2>Gal4*, *UAS-GFP /UAS-RhoGEF2-RNAi*
*hsFLP/UAS-dicer2; act>CD2>Gal4*, *UAS-GFP/UAS-scrib-RNAi; UAS-3HA-STAT92E^ΔNΔC^/UAS-RhoGEF2-RNAi*
**D,** *hsFLP/UAS-dicer2; UAS-scrib-RNAi/+; act>CD2>Gal4*, *UAS-GFP /UAS-RhoGEF2-RNAi*
**E,** *hsFLP/UAS-dicer2; act>CD2>Gal4*, *UAS-GFP/UAS-scrib-RNAi; UAS-3HA-STAT92E*^*ΔNΔC*^*/UAS-RhoGEF2-RNAi*

S1 Fig:

**A,** w; *ptc-Gal4*, *UAS-EGFP/+*
**B,** w; *UAS-dicer2/ptc-Gal4*, *UAS-EGFP; UAS-lgl-RNAi/+*
**C,** w; *ptc-Gal4*, *UAS-EGFP/+; UAS-Yki*^*M123*^*/+*

S2 Fig:

**A,** *hsFLP; UAS-scrib-RNAi/+; act>CD2>Gal4*, *UAS-GFP/UAS-dicer2*
**B,** *hsFLP; UAS-dicer2/+; act>CD2>Gal4*, *UAS-GFP/UAS-lgl-RNAi*

S3 Fig:

**A,** *w*, *sd-Gal4*, *UAS-EGFP/+*
**B,** *w*, *sd-Gal4*, *UAS-EGFP/+; UAS-dicer2/+; UAS-lgl-RNAi/+*
**C,** *hsFLP; 10xSTAT-GFP/UAS-scrib-RNAi; act>CD2>Gal4*, *UAS-RFP/UAS-dicer2*

S4 Fig:

**A–C,** *hsFLP/UAS-rpr;; act>CD2>Gal4*, *UAS-GFP/+*

S5 Fig:

**A,** *hsFLP; UAS-3HA-STAT92E*^*ΔNΔC*^*/+; act>CD2>Gal4*, *UAS-GFP/+*
**B,** *hsFLP; act>CD2>Gal4*, *UAS-GFP/UAS-scrib-RNAi; UAS-3HA-STAT92E*^*ΔNΔC*^*/UAS-dicer2*
**C,** *hsFLP/UAS-dicer2; UAS-scrib-RNAi/+; act>CD2>Gal4*, *UAS-GFP /UAS-RhoGEF2-RNAi*

### Immunohistochemistry and Image Analysis

Immunofluorescent stainings of imaginal discs were performed according to standard procedures for confocal microscopy as described previously [52]. The following antibodies were used: rabbit anti-aPKC (1:1000, Santa Cruz Biotechnology), rabbit anti-cleaved Caspase-3 (1:100, Cell Signaling), rabbit anti-Dcp-1 (1:100, Cell Signaling), mouse anti-armadillo N2 7A1 (1:40, Developmental Studies Hybridoma Bank [DSHB]), rat anti-DE-Cadherin DCAD2 (1:30, DSHB), mouse anti-Dlg 4F3 (1:40, DSHB), mouse anti-Fasciclin III 7G10 (1:15, DSHB), mouse anti-Integrin βPS CF.6G11 (1:100, DSHB), mouse anti-MMP1 (1:1:1 mixture of 3B8, 3A6 and 5H7 were diluted 1:10, DSHB), mouse anti-α-Spectrin 3A9 (1:50, DSHB), mouse anti-α-Tubulin AA4.4 (1:100, DSHB), rabbit anti-Lgl (1:100; a gift from J.A. Knoblich, IMBA, Vienna)[53], rabbit anti-phospho JNK pTPpY (1:100, Promega), and rabbit anti-Laminin-γ (1:100, abcam). Alexa Fluor 488, 546, and 633 (1:400, Molecular Probes) were used for secondary antibodies. F-actin was stained by Alexa Fluor 546 Phalloidin (1:50, Molecular Probes). Images were captured on a Zeiss LSM 510 confocal microscope or on an Olympus FV1200 confocal microscope. 3-D reconstructions of confocal z-stack images were rendered with ImageJ 3-D Viewer, an ImageJ plugin (B. Schmid, 2007). Signal intensities were plotted with Interactive 3-D Surface Plot, an ImageJ plugin (K.U. Barthel, 2004).

### Transmission Electron Microscopy

The sample preparations were performed according to standard procedures for TEM as described previously [54]. Dissected imaginal discs were fixed in a solution of 2.5% glutaraldehyde and 2% paraformaldehyde buffered with 0.1 M sodium cacodylate to pH7.4. They were post-fixed with 1% osmium tetroxide in the same buffer, stained en bloc with 0.5% uranyl acetate in distilled water, dehydrated in ethanol, and embedded in Epon. Ultra-thin sections (70 nm) were stained with 2% uranyl acetate solution and Reynold’s lead citrate solution. Images were obtained with a VELETA CCD Camera (Olympus Soft Imaging Solutions) mounted on a JEM 1010 transmission electron microscope (JEOL).

### Quantification of Tumorigenesis Occurrence Ratio

Tumorigenesis was induced by random expression of nTSG-RNAi using the heat-shock activated flip-out Gal4-UAS system, and the occurrence ratio of tumorigenesis was counted 4 to 7 d after clone induction. Each occurrence of tumorigenesis includes a dysplastic cell mass deviating from the disc-proper epithelial layer with a diameter larger than four cells. The percentages of tumor growths were quantified from more than three independent experiments for each genotype. The numerical data used in Fig 8A and 8B are included in S1 Data.

### Statistical Analysis

Two-tailed unpaired *t* tests assuming equal variances were performed for all statistical analyses. *p*-Value < 0.001 was considered statistically significant for all analyses.

## Supporting Information

S1 Data Excel spreadsheet containing, in separate sheets, the underlying numerical data for Fig 8A and 8B. (XLSX)

S1 Fig Cell-competition-induced apoptosis in nTSG knockdown cells. (**A–B**) Confocal images show wing discs dissected from indicated genotypes. Regions expressing *ptc-Gal4* were labeled by GFP expression (green). Apoptotic cells were labeled with anti-cleaved Caspase-3 antibody (red) in (**A**) and (**B**). Nuclei were labeled with DAPI (blue). A white dotted line marks the boundaries between the wing pouch and hinge regions in (**C**). Scale bars represent 50 μm in (**A–B**) and 100 μm in (**C**).
(TIF)

S2 Fig JNK activity and MMP1 expression are up-regulated in nTSG-knockdown-induced tumors. (**A**) Mosaic wing disc six days after induction of random *scrib-RNAi* expression stained for phosphorylated JNK (red). (**B**) Mosaic wing disc 6 d after induction of random *lgl-RNAi* expression stained for MMP1 (red). RNAi-expressing cells were labeled by GFP expression (green). Nuclei were labeled with DAPI (blue). Scale bars represent 50 μm.
(TIF)

S3 Fig nTSG knockdown induces site-specific tumorigenesis in the wing disc. (**A**) Wing disc of third instar larva with *scalloped-Gal4* (*sd-Gal4*) stained for MMP1 (red). *sd-Gal4*-expressing regions are labeled by GFP expression (green). (**B**) Wing disc of third instar larva expressing *lgl-RNAi* in the *sd-Gal4*-expressing regions (green), stained for MMP1 (red). (**C**) Wing disc with mosaic clones expressing RFP and *scrib-RNAi* 5 d after clone induction. RNAi-expressing cells were labeled by RFP expression (red). 10xSTAT-GFP, green. Nuclei were labeled with DAPI (blue). Arrows indicate medial fold of dorsal hinge region. White dotted lines mark the boundaries between the wing pouch and hinge regions. Scale bars represent 100 μm.
(TIF)

S4 Fig Apoptotic cells are extruded towards the basal side in both coldspots and hotspots. (**A**) Vertical section of a wing disc with mosaic clones expressing GFP and a pro-apoptotic gene, *Reaper* (*Rpr*), 24 h after clone induction stained for Laminin-γ (magenta). (**B–C**) Magnifications of coldspot (**B**) and hotspot (**C**) regions. Arrows indicate basally extruded apoptotic cells. Nuclei were labeled with DAPI (blue). Scale bars represent 50 μm in (**A**) and 10 μm in (**B**) and (**C**).
(TIF)

S5 Fig JAK/STAT signaling activity is not involved in the delamination direction of nTSG-knockdown cells. (**A**) Vertical section of a wing disc with mosaic clones expressing GFP and a constitutively active form of STAT92E, 2 d after clone induction, stained for α-tubulin (magenta). Lower panels: magnifications of the box indicated in the upper panel. (**B**) Vertical section of a wing disc with mosaic clones (expressing GFP, green) co-expressing *scrib-RNAi* and a constitutively active form of STAT92E 2 d after clone induction, stained for α-tubulin (magenta). Lower panels: magnifications of the box indicated in the upper panel. Arrowheads indicate basally extruded clones. (**C**) Wing disc with mosaic clones co-expressing *scrib-RNAi* and *RhoGEF2-RNAi* 3 d after clone induction. RNAi-expressing cells were labeled by RFP expression (red). 10xSTAT-GFP, green. A white dotted line marks the boundaries between the wing pouch and hinge regions. Nuclei were labeled with DAPI (blue). Scale bars represent 10 μm in (**A**) and (**B**) and 50 μm in (**C**).
(TIF)

## Abbreviations

AP: anterior-posterior
aPKC: atypical protein kinase C
dlg: discs large
ECM: extracellular matrix
FLP: flippase
FRT: flippase recognition target
IL-6: Interleukin-6
IR: ionizing radiation
JAK/STAT: Janus kinase/signal transducer and activator of transcription
lgl: *lethal giant* larvae
MMP1: matrix metalloproteinase-1
MTs: microtubules
nTSG: neoplastic tumor-suppressor gene
pJNK: phosphorylated c-Jun N-terminal kinase
RNAi: RNA interference
scrib: scribble
TEM: transmission electron micrographs
TNF: tumor necrosis factor
Upd: Unpaired
