# Supplementary figures and images for: Epithelial Tumors Originate in Tumor Hotspots, a Tissue-Intrinsic Microenvironment
Source: PLoS Biol. 2016 Sep 1;14(9):e1002537. doi: 10.1371/journal.pbio.1002537 (PMC5008749; doi:10.1371/journal.pbio.1002537)

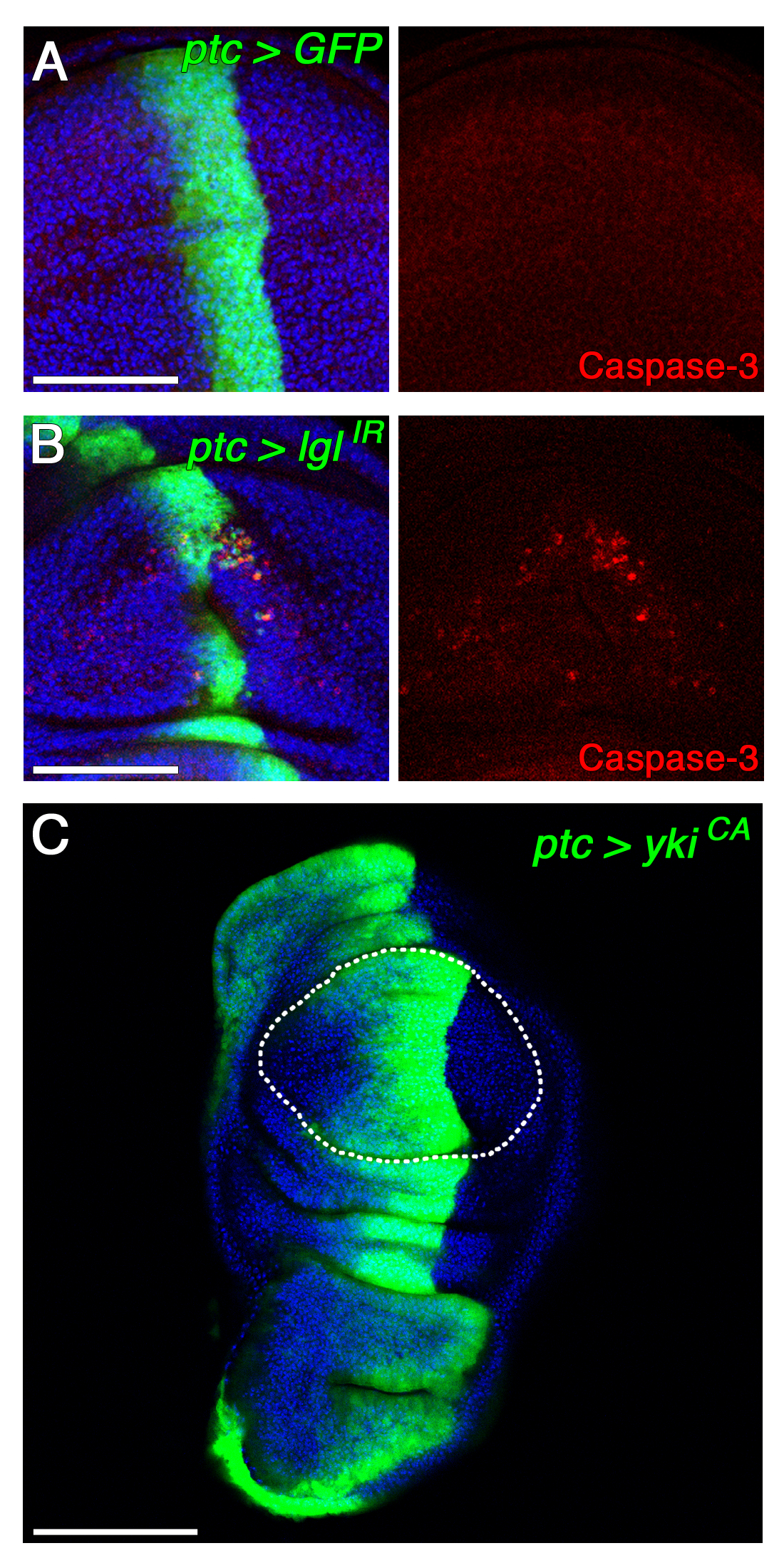

Supplement: S1 Fig — (A–B) Confocal images show wing discs dissected from indicated genotypes. Regions expressing ptc-Gal4 were labeled by GFP expression (green). Apoptotic cells were labeled with anti-cleaved Caspase-3 antibody (red) in (A) and (B). Nuclei were labeled with DAPI (blue). A white dotted line marks the boundaries between the wing pouch and hinge regions in (C). Scale bars represent 50 μm in (A–B) and 100 μm in (C). (TIF) [file pbio.1002537.s002.tif]

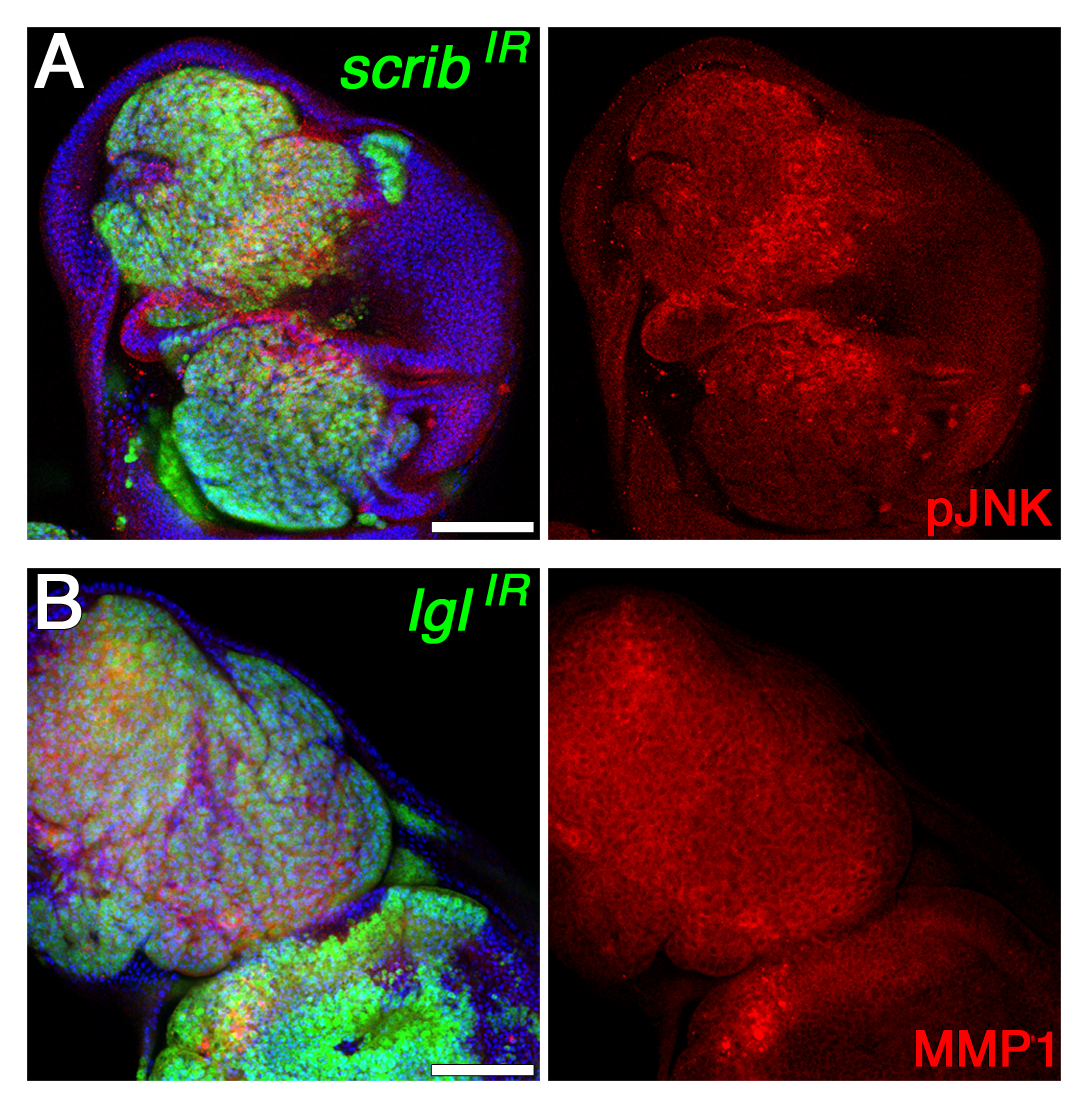

Supplement: S2 Fig — (A) Mosaic wing disc six days after induction of random scrib-RNAi expression stained for phosphorylated JNK (red). (B) Mosaic wing disc 6 d after induction of random lgl-RNAi expression stained for MMP1 (red). RNAi-expressing cells were labeled by GFP expression (green). Nuclei were labeled with DAPI (blue). Scale bars represent 50 μm. (TIF) [file pbio.1002537.s003.tif]

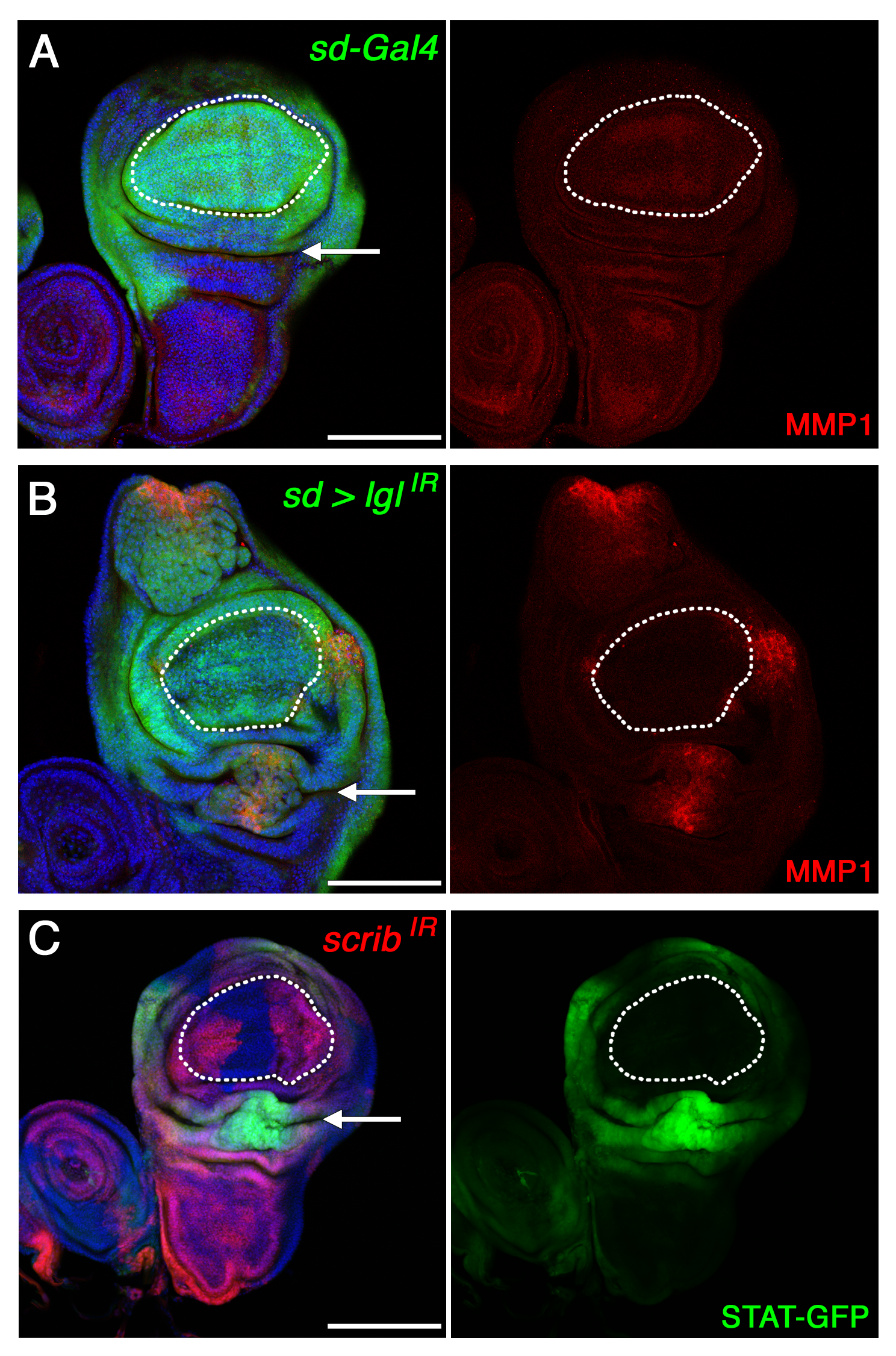

Supplement: S3 Fig — (A) Wing disc of third instar larva with scalloped-Gal4 (sd-Gal4) stained for MMP1 (red). sd-Gal4-expressing regions are labeled by GFP expression (green). (B) Wing disc of third instar larva expressing lgl-RNAi in the sd-Gal4-expressing regions (green), stained for MMP1 (red). (C) Wing disc with mosaic clones expressing RFP and scrib-RNAi 5 d after clone induction. RNAi-expressing cells were labeled by RFP expression (red). 10xSTAT-GFP, green. Nuclei were labeled with DAPI (blue). Arrows indicate medial fold of dorsal hinge region. White dotted lines mark the boundaries between the wing pouch and hinge regions. Scale bars represent 100 μm. (TIF) [file pbio.1002537.s004.tif]

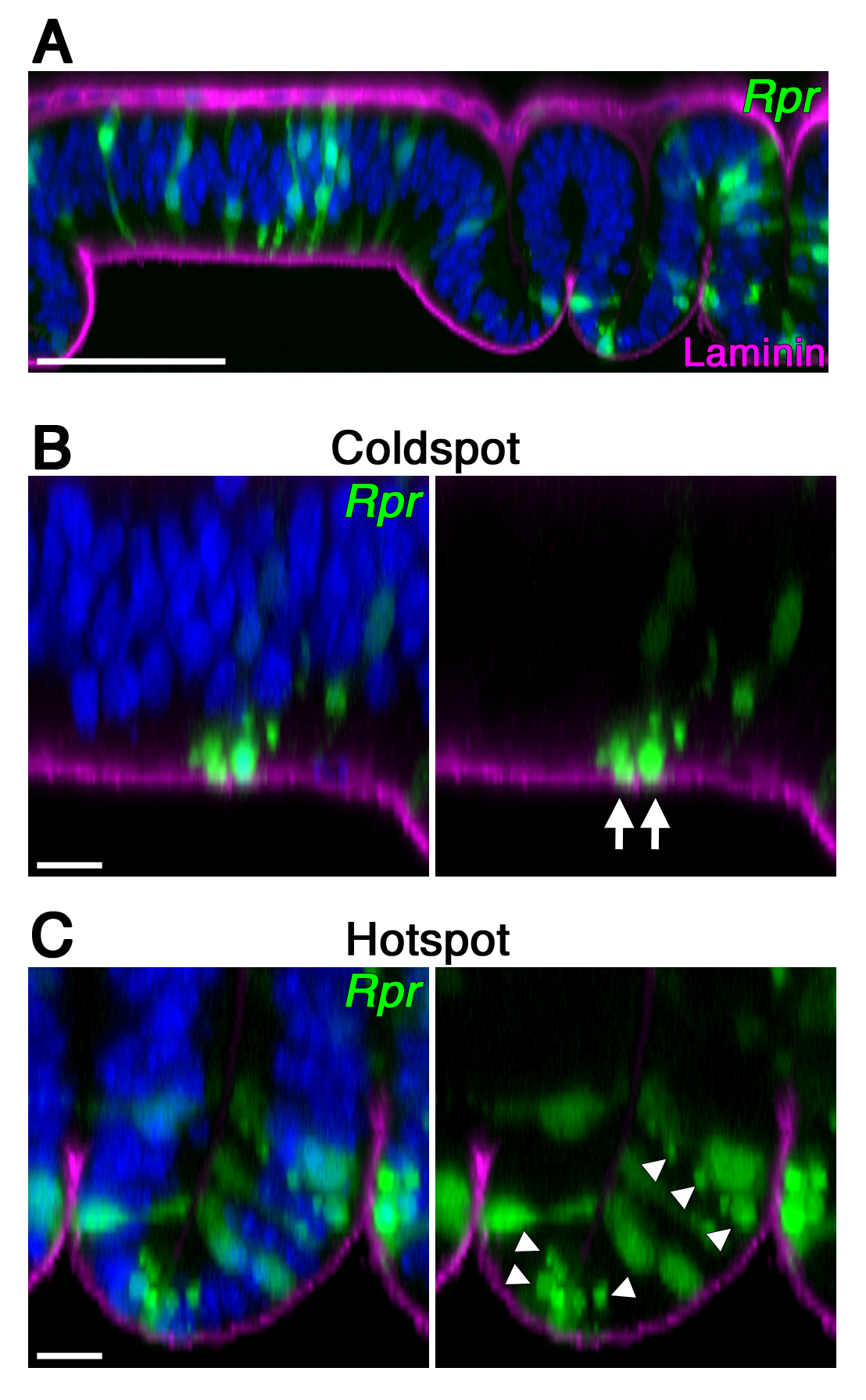

Supplement: S4 Fig — (A) Vertical section of a wing disc with mosaic clones expressing GFP and a pro-apoptotic gene, Reaper (Rpr), 24 h after clone induction stained for Laminin-γ (magenta). (B–C) Magnifications of coldspot (B) and hotspot (C) regions. Arrows indicate basally extruded apoptotic cells. Nuclei were labeled with DAPI (blue). Scale bars represent 50 μm in (A) and 10 μm in (B) and (C). (TIF) [file pbio.1002537.s005.tif]

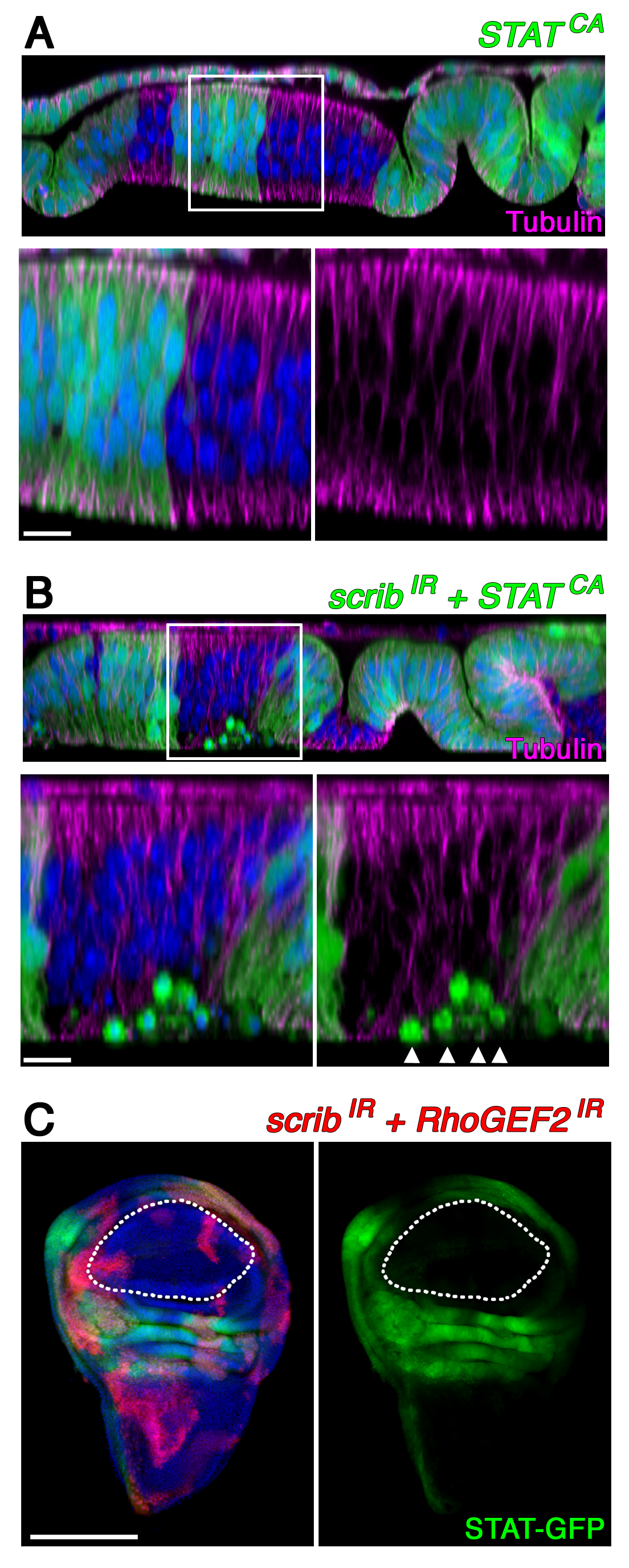

Supplement: S5 Fig — (A) Vertical section of a wing disc with mosaic clones expressing GFP and a constitutively active form of STAT92E, 2 d after clone induction, stained for α-tubulin (magenta). Lower panels: magnifications of the box indicated in the upper panel. (B) Vertical section of a wing disc with mosaic clones (expressing GFP, green) co-expressing scrib-RNAi and a constitutively active form of STAT92E 2 d after clone induction, stained for α-tubulin (magenta). Lower panels: magnifications of the box indicated in the upper panel. Arrowheads indicate basally extruded clones. (C) Wing disc with mosaic clones co-expressing scrib-RNAi and RhoGEF2-RNAi 3 d after clone induction. RNAi-expressing cells were labeled by RFP expression (red). 10xSTAT-GFP, green. A white dotted line marks the boundaries between the wing pouch and hinge regions. Nuclei were labeled with DAPI (blue). Scale bars represent 10 μm in (A) and (B) and 50 μm in (C). (TIF) [file pbio.1002537.s006.tif]
